# Supplementary material for: Sitagliptin Mitigates Diabetic Nephropathy in a Rat Model of Streptozotocin-Induced Type 2 Diabetes: Possible Role of PTP1B/JAK-STAT Pathway
Source: Int J Mol Sci. 2023 Mar 31;24(7):6532. doi: 10.3390/ijms24076532 (PMC10095069; doi:10.3390/ijms24076532)
Supplement: Supplementary file 1 [file ijms-24-06532-s001.zip › Figure S1 (Original unprocessed immunoblots for all proteins of interest).pdf]

Figure S1: Original unprocessed immunoblot for all Proteins of interest

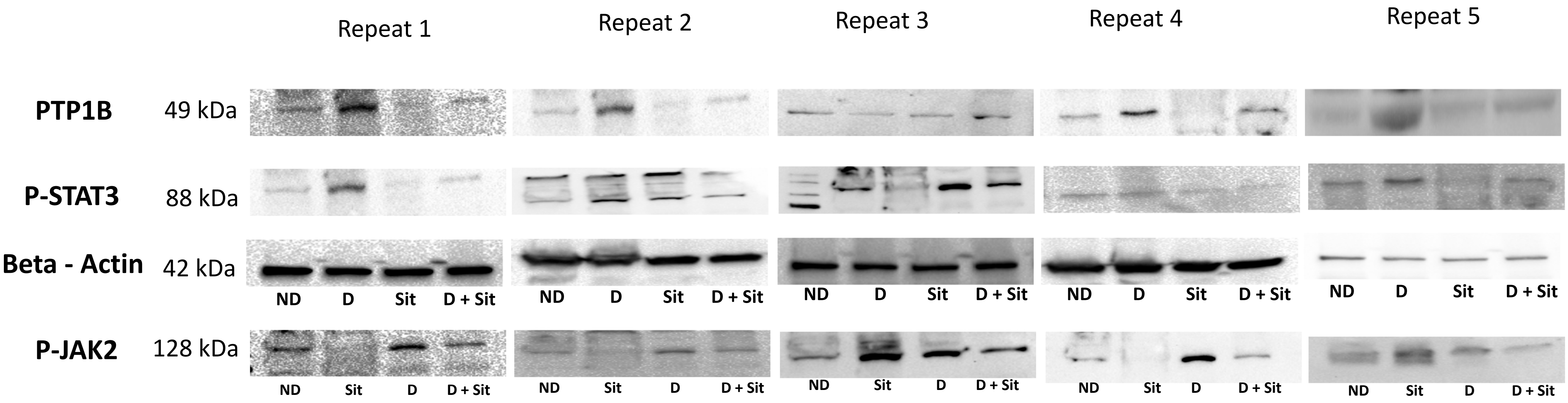

PTP1B

ND D Sit D + Sit

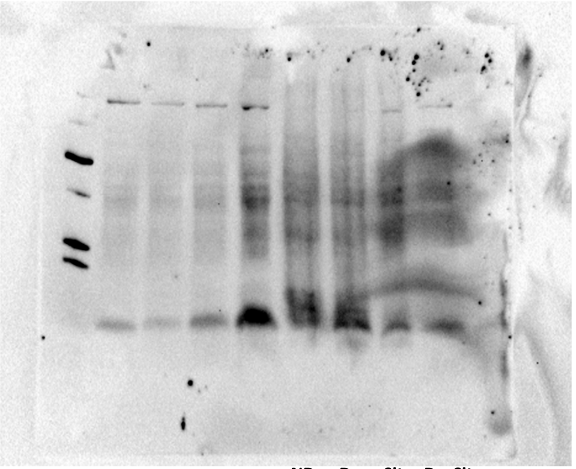

ND D Sit D + Sit

ND D Sit D + Sit

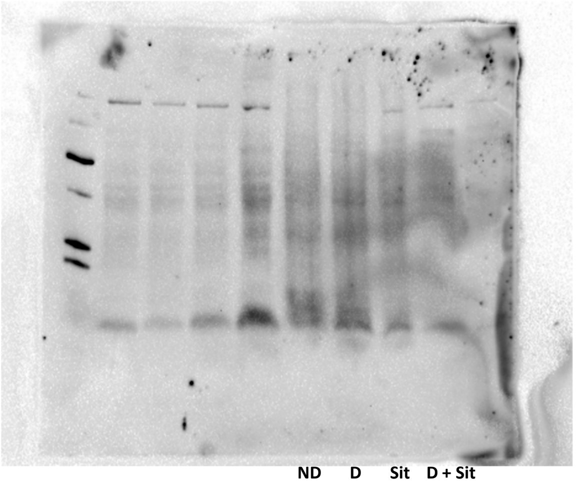

ND D Sit D + Sit

ND D Sit D + Sit

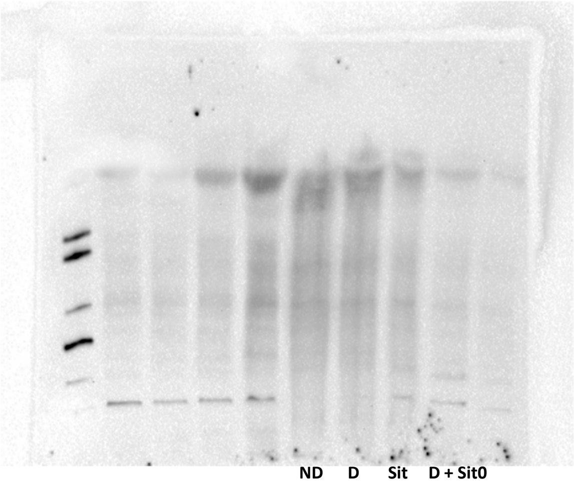

ND D Sit D + Sit0

ND D Sit D + Sit

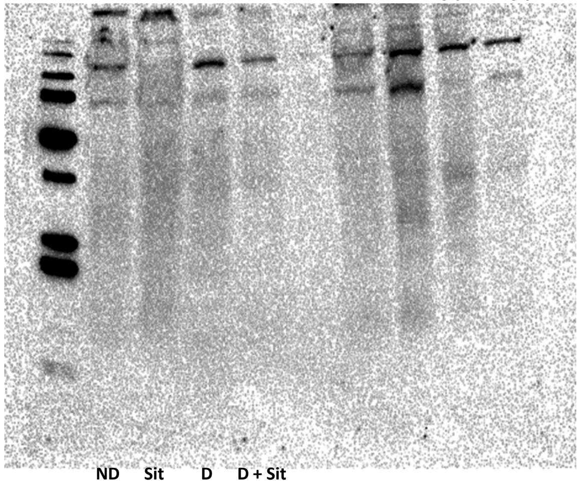

ND Sit D D + Sit

ND D Sit D + Sit

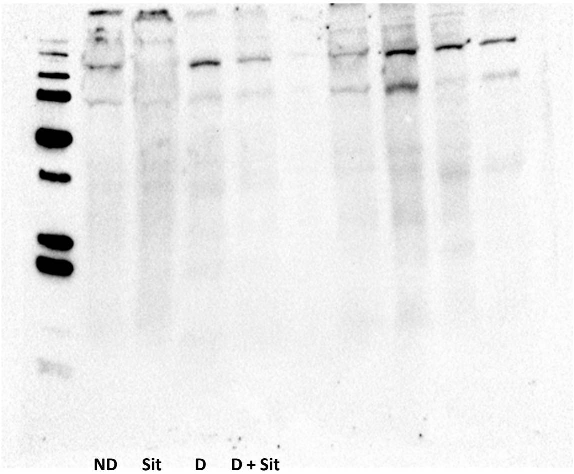

ND Sit D D + Sit

Beta -actin

ND D Sit D + Sit

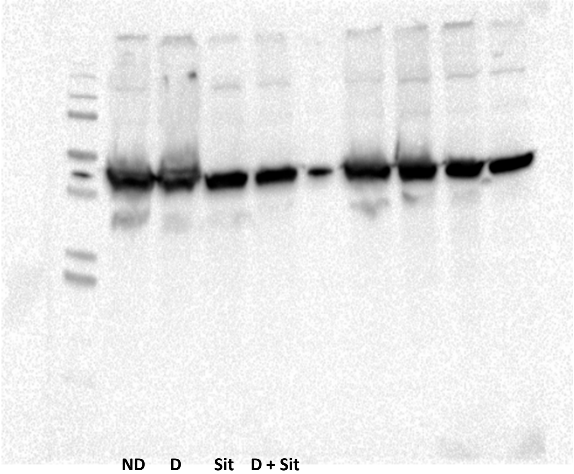

ND D Sit D + Sit

ND D Sit D + Sit

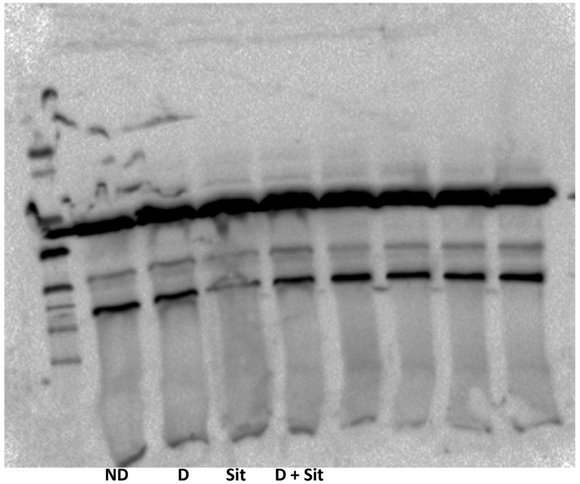

ND D Sit D + Sit

ND D Sit D + Sit

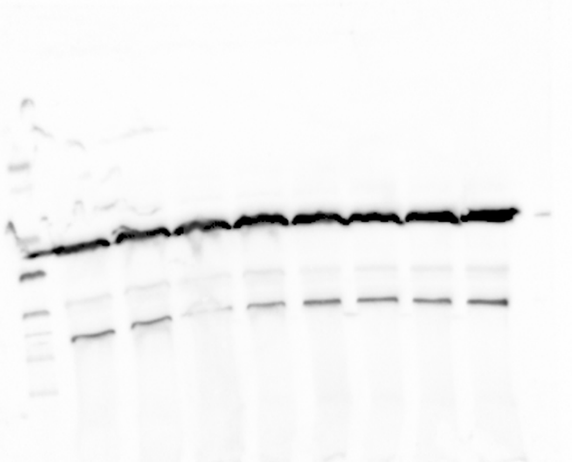

ND D Sit D + Sit

ND D Sit. D + Sit

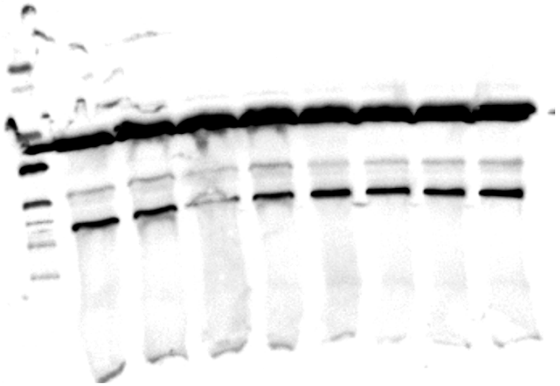

ND D Sit. D + Sit

ND D Sit D + Sit

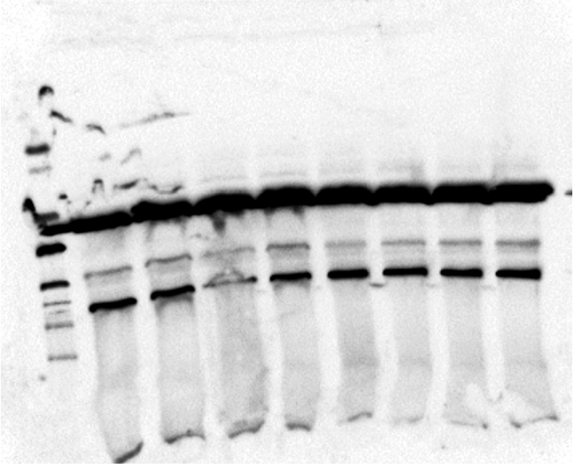

ND D Sit. D + Sit

P-JAK2

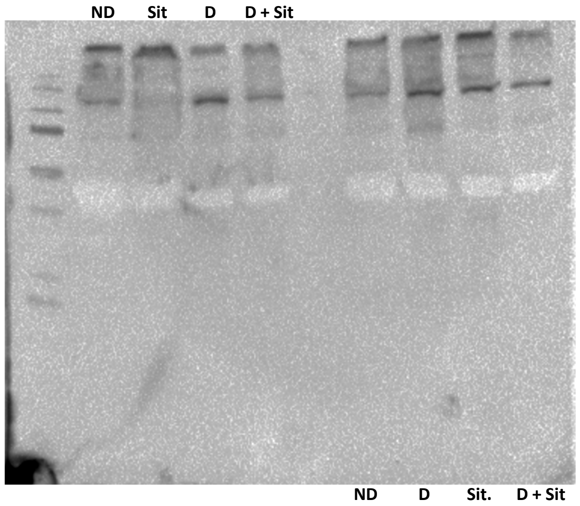
ND
Sit
D
D + Sit
ND
D
Sit.
D + Sit

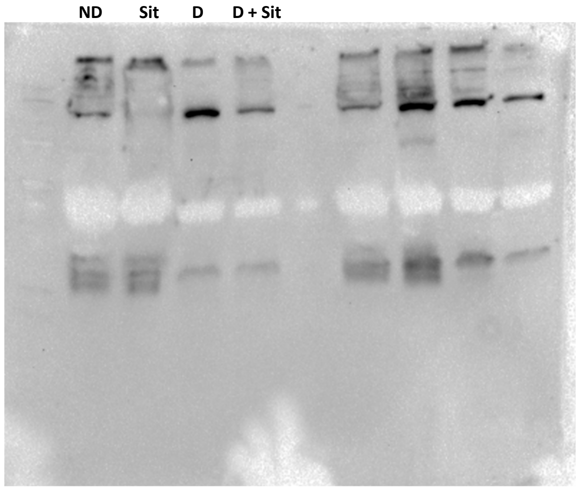
ND
Sit
D
D + Sit

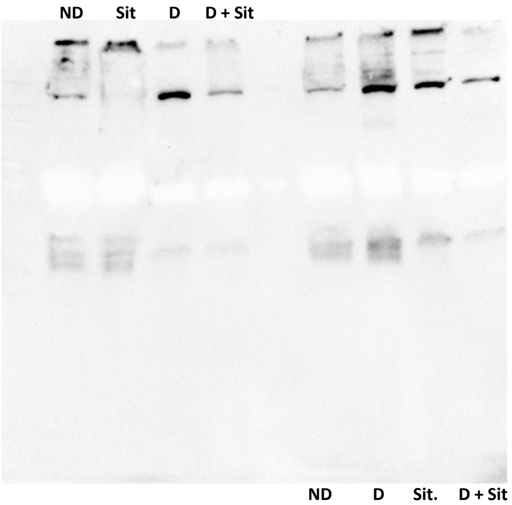
ND
Sit
D
D + Sit
ND
D
Sit.
D + Sit

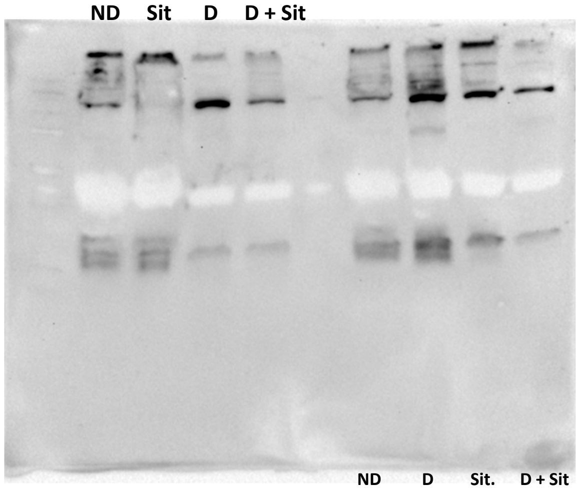
ND
Sit
D
D + Sit
ND
D
Sit.
D + Sit

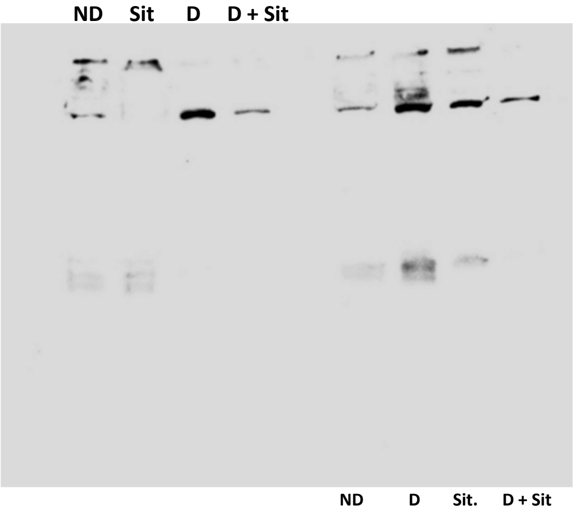
ND
Sit
D
D + Sit
ND
D
Sit.
D + Sit

P-STAT3

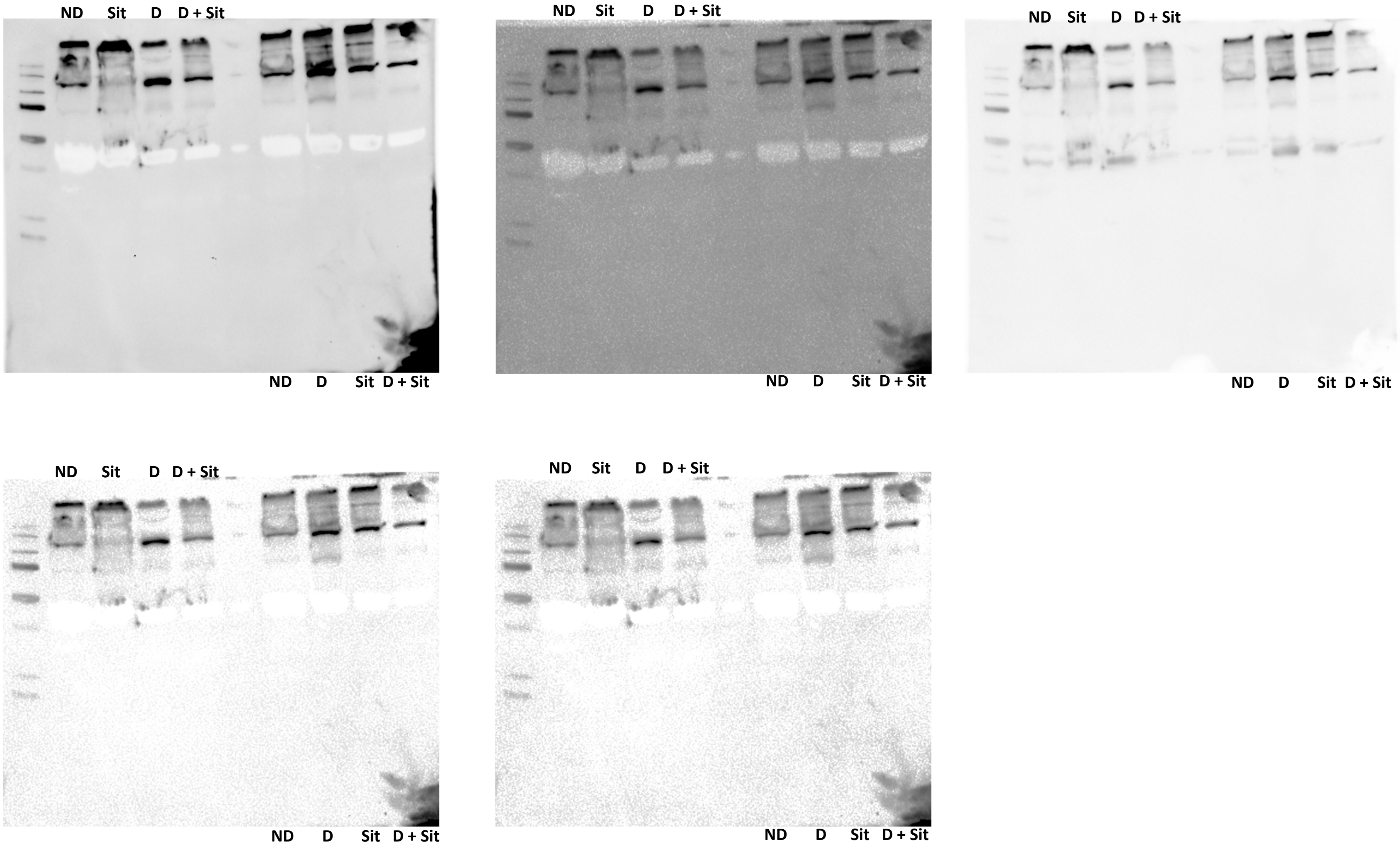
ND Sit D D + Sit
ND D Sit D + Sit
ND Sit D D + Sit
ND D Sit D + Sit
ND Sit D D + Sit
ND D Sit D + Sit
ND Sit D D + Sit
ND D Sit D + Sit
ND Sit D D + Sit
ND D Sit D + Sit
