# Supplementary material for: Sitagliptin Mitigates Diabetic Nephropathy in a Rat Model of Streptozotocin-Induced Type 2 Diabetes: Possible Role of PTP1B/JAK-STAT Pathway
Source: Int J Mol Sci. 2023 Mar 31;24(7):6532. doi: 10.3390/ijms24076532 (PMC10095069; doi:10.3390/ijms24076532)
Supplement: Supplementary file 1 [file ijms-24-06532-s001.zip › Table S1 (Original data for all biochemical and molecular parameters).pdf]

Table S1 (A). Original data for the effects of sitagliptin on DN biomarkers in STZ-induced diabetic rats (glucose levels (mg/dl)).

	Normal Control	Normal Sitagliptin	STZ Control	STZ Sitagliptin
Repeat no. 1	3.69000	4.74000	7.39000	5.46000
Repeat no. 2	4.87000	3.56000	5.34000	4.04000
Repeat no. 3	3.1000	4.04000	13.32000	4.97000
Repeat no. 4	2.1100	3.33000	11.29000	6.54000
Repeat no. 5	5.31000	4.26000	8.73000	0.34000
Repeat no. 6	2.55000	3.55000	7.05000	3.88000
Repeat no. 7	4.13000	2.50000	7.58000	3.82000
Repeat no. 8	4.03000	5.84000	13.000	5.02000

Table S1 (B). Original data for the effects of sitagliptin on DN biomarkers in STZ-induced diabetic rats (kidney weight (g)).

	Normal Control	Normal Sitagliptin	STZ Control	STZ Sitagliptin
Repeat no. 1	2.4	2.29	2.94	2
Repeat no. 2	1.45	1.91	2.25	1.82
Repeat no. 3	1.47	1.9	2.17	1.7
Repeat no. 4	2.09	2	2.4	1.45
Repeat no. 5	1.56	1.5	3.5	2.02
Repeat no. 6	2.04	2.53	3.04	1.97
Repeat no. 7	1.62	2.22	3.6	1.98
Repeat no. 8	2.015	1.85	2.55	2.04

Table S1 (C). Original data for the effects of sitagliptin on DN biomarkers in STZ-induced diabetic rats (kidney/body weight Ratio (%)).

	Normal Control	Normal Sitagliptin	STZ Control	STZ Sitagliptin
Repeat no. 1	0.773398	0.85000	1.10000	0.72000
Repeat no. 2	0.53891	0.61000	0.86000	0.59000
Repeat no. 3	0.45000	0.71000	0.80000	0.73000
Repeat no. 4	0.695221	0.84000	0.94000	0.66000
Repeat no. 5	0.42250	0.69000	1.21000	0.72000
Repeat no. 6	0.64210	0.768000	1.31000	0.68000
Repeat no. 7	0.48811	0.795000	1.22000	0.65000
Repeat no. 8	0.58120	0.65900	0.89000	0.71000

Table S1 (D). Original data for the effects of sitagliptin on DN biomarkers in STZ-induced diabetic rats (urea (mg/dl)).

	Normal Control	Normal Sitagliptin	STZ Control	STZ Sitagliptin
Repeat no. 1	6.76	9.36	59.07	50.58
Repeat no. 2	24.23	6.37	58.78	14.29
Repeat no. 3	6.56	13.9	38.71	10.42
Repeat no. 4	14	11.87	37.74	11.97
Repeat no. 5	6.37	7.53	47.39	54.54
Repeat no. 6	13.61	8.88	56.56	70.08
Repeat no. 7	6.02	6.5129	35.29	15.60243
Repeat no. 8	17.822	12.78	64.0392	55.0328

Table S1 (E). Original data for the effects of sitagliptin on DN biomarkers in STZ-induced diabetic rats (BUN (mg/dl)).

	Normal Control	Normal Sitagliptin	STZ Control	STZ Sitagliptin
Repeat no. 1	3.1500	4.3700	27.5700	23.6000
Repeat no. 2	11.3100	2.9700	27.4300	6.6700
Repeat no. 3	3.0600	6.4900	18.0600	4.8600
Repeat no. 4	6.5300	5.5400	17.6100	5.5900
Repeat no. 5	2.9700	3.5100	22.1200	25.4500
Repeat no. 6	6.3500	4.1400	26.5900	28.9700
Repeat no. 7	6.1200	3.5500	18.9400	18.42000
Repeat no. 8	5.000	5.4500	27.2399	18.18123

Table S1 (F). Original data for the effects of sitagliptin on DN biomarkers in STZ-induced diabetic rats (creatinine (IU/L)).

	Normal Control	Normal Sitagliptin	STZ Control	STZ Sitagliptin
Repeat no. 1	0.3200	0.1300	0.8700	0.1600
Repeat no. 2	0.3600	0.1600	0.3900	0.1000
Repeat no. 3	0.2100	0.2700	0.7600	0.6800
Repeat no. 4	0.1900	0.2500	0.2500	0.2000
Repeat no. 5	0.3600	0.3100	0.8400	0.3500
Repeat no. 6	0.2700	0.2700	0.7400	0.3700

Repeat no. 7	0.3000	0.2200	0.700	0.2500
Repeat no. 8	0.2500	0.3900	0.5800	0.3800

Table S1 (G). Original data for the effects of sitagliptin on interleukin-6 (IL-6) plasma levels (pg/ml) in STZ-induced diabetic rats.

	Normal Control	Normal Sitagliptin	STZ Control	STZ Sitagliptin
Repeat no. 1	12.49000	7.46000	24.20000	10.4300
Repeat no. 2	11.37000	7.72000	19.24385	9.50000
Repeat no. 3	9.85000	7.93000	20.06809	7.72000
Repeat no. 4	13.55000	6.97000	20.41317	10.3600
Repeat no. 5	11.23000	8.51000	19.88000	9.31000
Repeat no. 6	10.49000	6.13000	19.54192	9.75000
Repeat no. 7	10.51000	9.17000	19.92170	8.78000
Repeat no. 8	11.48000	6.23000	22.34656	9.42000

Table S1 (H). Original data for the effects of sitagliptin on tumour necrosis factor alpha (TNF-α) plasma levels (pg/mg protein) in STZ-induced diabetic rats.

	Normal Control	Norrmal Sitagliptin	STZ Control	STZ Sitagliptin
Repeat no. 1	235.32999	174.10289	352.82000	111.31208
Repeat no. 2	206.69583	215.04000	316.62000	114.09420
Repeat no. 3	249.88022	211.24000	376.40620	120.22805
Repeat no. 4	200.33092	194.30352	300.96000	120.41749
Repeat no. 5	250.33415	222.87000	321.02098	148.41028
Repeat no. 6	278.88159	253.57596	326.36000	124.26572
Repeat no. 7	252.10496	210.34945	321.13000	122.63712
Repeat no. 8	203.68390	204.48000	315.32000	117.68842

Table S1 (I). Original data for the effects of sitagliptin on phosphorylated Janus kinase (P-JAK2) protein expression (fold of induction) in STZ-induced diabetic rats.

	Normal Control	Normal Sitagliptin	STZ Control	STZ Sitagliptin
Repeat no. 1	1.000	1.13759	8.37837	2.20720
Repeat no. 2	1.010	1.03059	8.30083	2.00720
Repeat no. 3	1.090	1.00759	8.95378	2.57207
Repeat no. 4	1.000	1.11113	8.00000	2.00002
Repeat no. 5	1.000	1.00005	8.00337	2.00720
Repeat no. 6	1.09	1.00700	8.00097	2.00010

Repeat no. 7	1.000	1.00030	8.00030	2.00007
Repeat no. 8	1.000	1.00007	8.00007	2.00007

Table S1 (J). Original data for the effects of sitagliptin on the phosphorylated signal transducer and activator of transcription (P-STAT3) protein expression (fold of induction) in STZ-induced diabetic rats.

	Normal Control	Normal Sitagliptin	STZ Control	STZ Sitagliptin
Repeat no. 1	1.000	1.219032	2.157096	1.38870
Repeat no. 2	1.000	1.119032	2.957096	0.918387
Repeat no. 3	1.100	1.217032	2.157096	0.98387
Repeat no. 4	1.000	1.29000	2.570007	1.800080
Repeat no. 5	1.000	1.001010	2.97000	0.90000
Repeat no. 6	1.000	1.17000	2.111154	0.82299
Repeat no. 7	1.000	1.21000	2.115000	1.30010
Repeat no. 8	1.010	1.11000	2.910010	0.90000

Table S1 (K). Original data for the effects of sitagliptin on protein tyrosine phosphatase 1B (PTP1B) protein expression (fold of induction) in STZ-induced diabetic rats.

	Normal Control	Normal Sitagliptin	STZ Control	STZ Sitagliptin
Repeat no. 1	1	1.014251	5.97235094	1.44748224
Repeat no. 2	1	1.02451	5.57235094	1.4474224
Repeat no. 3	1	1.224251	5.77235	1.04748224
Repeat no. 4	1	1.014251	5.97235094	1.035578821
Repeat no. 5	1	1.02451	5.57235094	1.398400444
Repeat no. 6	1	1.224251	5.77235	1.4474224
Repeat no. 7	1	1.111002323	5.5550032	1.000224
Repeat no. 8	1	1.221100432	5.884344	1.00780022
